# Double Cluster Heads Model for Secure and Accurate Data Fusion in Wireless Sensor Networks

**DOI:** 10.3390/s150102021

**Published:** 2015-01-19

**Authors:** Jun-Song Fu, Yun Liu

**Affiliations:** School of Electronic and Information Engineering, Key Laboratory of Communication and Information Systems, Beijing Municipal Commission of Education, Beijing Jiaotong University, Beijing 100044, China; E-Mail: 12120067@bjtu.edu.cn

**Keywords:** Double Cluster Heads Model, clustering, data fusion, security, accuracy

## Abstract

Secure and accurate data fusion is an important issue in wireless sensor networks (WSNs) and has been extensively researched in the literature. In this paper, by combining clustering techniques, reputation and trust systems, and data fusion algorithms, we propose a novel cluster-based data fusion model called Double Cluster Heads Model (DCHM) for secure and accurate data fusion in WSNs. Different from traditional clustering models in WSNs, two cluster heads are selected after clustering for each cluster based on the reputation and trust system and they perform data fusion independently of each other. Then, the results are sent to the base station where the dissimilarity coefficient is computed. If the dissimilarity coefficient of the two data fusion results exceeds the threshold preset by the users, the cluster heads will be added to blacklist, and the cluster heads must be reelected by the sensor nodes in a cluster. Meanwhile, feedback is sent from the base station to the reputation and trust system, which can help us to identify and delete the compromised sensor nodes in time. Through a series of extensive simulations, we found that the DCHM performed very well in data fusion security and accuracy.

## 1. Introduction

Wireless sensor networks (WSNs) are composed of a large number of wireless connected sensor nodes, which are strictly limited by power supply, computational capability, storage resources and communication bandwidth. To our knowledge, WSNs are increasingly used in many fields, such as military surveillance, agriculture monitoring and forest fire detection. After being deployed manually or scattered by aircrafts, the sensor nodes automatically construct a network connected with the base station, which is controlled by the users directly. Each sensor node senses the surrounding environment and then transfers the data to the base station by a one-hop or multi-hop method.

In WSNs, saving energy is one of the most important issues. Data fusion can reduce energy consumption significantly because sensor nodes might generate significant redundant data and it is a waste of energy to transmit all the data. Recognizing that in-network computation would generate less energy consuming than communication, we can save the energy by fusing the data and transmitting the data fusion results instead of the original data. Multi-sensor data fusion algorithms have been extensively researched [1]. Bayesian data fusion [1] is one of the most important data fusion algorithms based on probability. In Double Cluster Heads Model (DCHM), we use it to fuse data. In large WSNs, clustering is an important management strategy, which has the natural ability to facilitate data fusion in WSNs.

Another essential issue is security. Traditionally, cryptography is used to provide a solution for secure and reliable data fusion problem [2,3]. However, because of collaborative data processing problem and data authentication problem [4], it is not sufficient to protect network by cryptography only. As a result, reputation and trust systems originated from sociology are introduced into WSNs. In this paper, we define reputation and trust as follows [5,6]:

*Reputation*: perception that an agent creates about another agent's intention and norms, through direct and indirect observation of its' past actions.
*Trust*: a subjective expectation an agent has about another's future behavior with respect to a specific action.

In a WSNs' reputation and trust system, the actions of every sensor node are observed by other sensor nodes in an attempt to evaluate its trustworthiness. However, for a large WSN, it is very difficult to build and maintain a reputation and trust system for the whole network, because the time delay is too large and it would take a lot of energy, which is strictly limited in WSNs. So in this paper a reputation and trust system is built and maintained by each small cluster.

We find that, clustering, reputation and trust systems, and data fusion have one thing in common, *i.e.*, locality, which allows them to supplement each other. Therefore, we propose a novel, cluster-based model by combing these three techniques, which is called Double Cluster Heads Model (DCHM) for secure and accurate data fusion in wireless sensor networks. Different from traditional clustering models, two cluster heads are selected for a cluster based on the reputation and trust system in DCHM and they perform data fusion independently of each other. It is highly possible that some sensor nodes in a WSN are compromised and selected as cluster heads. A compromised sensor node will obstruct data fusion by several attacks, such as jamming, message dropping, information falsifying and so on. We assume that the compromised cluster heads output a randomly generated result to mislead the users. Therefore, the outputs of two cluster heads in a cluster may be obviously different if one or two of them are compromised. Note that, not only the adversary or malicious sensor nodes can be detected and added to the blacklist, but also the breakdown sensor nodes can't monitor the surrounding accurately. From this view, the framework in this paper performs better than cryptography. We validate the performance of our model by several experiments. Simulations illustrates that DCHM performs very well in secure and accurate data fusion.

Our contributions are summarized as follows:

(A) We integrate clustering algorithm, reputation and trust systems, and data fusion together to provide a solution for secure and accurate data fusion. It is an interesting research direction in the field of data fusion in WSNs.
(B) The security of data fusion improved by selecting credible sensor nodes as cluster heads; the accuracy is improved by deleting the outliers and fusing credible data.
(C) Compared with traditional ones, the reputation and trust system in our paper converged faster and a reasonable explanation is that the feedback to the system makes contributions to the convergence time.

The rest of this paper is organized as follows. Section 2 reviews the related work, and Section 3 describes the assumptions and the thread model. We discuss the DCHM in Section 4. Section 5 presents the simulation results and Section 6 gives concludes for this paper.

## 2. Related Work

Several problems related to our protocol have been widely researched in wireless sensor networks.

### 2.1. Clustering Algorithms for Wireless Sensor Networks

We will give some published distributed algorithms for clustering WSNs. As far as we know, linked cluster algorithm (LCA) [7,8] is one of the earliest clustering algorithms. In LCA, cluster heads form a backbone network and connect with all the sensor nodes in its cluster directly. Hierarchical control clustering algorithm [9] forms a multi-tier hierarchical cluster structure. When clustering WSNs, the authors in [10] argued that it was very unwise to ignore the geographical information of the sensor nodes, especially for a large WSN. Therefore, they proposed GS^3^ clustering algorithm, which used geographical radius of cluster instead of logical radius. Low energy adaptive clustering hierarchy (LEACH) [11] is another popular clustering algorithm for WSNs. LEACH forms clusters based on the received signal strength and uses the cluster heads as routers to the base station. It naturally facilitates the creation of a reputation system locally in a cluster and data fusion in WSNs.

### 2.2. Reputation and Trust for Wireless Sensor Networks

In WSNs, cryptography performs very well in providing data confidentiality, data integrity, sensor nodes certification and so on. It is definitely that all these techniques are needed to construct a secure WSN. However, cryptography alone can't ensure the safety of data fusion in WSNs [4,12].

First, the sensor nodes in a common WSN are envisioned to be cheap and therefore very unlikely to be equipped with tamper-proof hardware. As a result, sensor nodes can be compromised by an attacker and the cryptography materials are recovered. The faked sensor nodes with the cryptography can communicate with other sensors and then attack data fusion process by generating some bogus data, which can mislead data fusion results.

Second, hardware failure in WSNs is another important issue that can't be solved by cryptography. Most sensor nodes are made of cheap hardware and it is common that some sensor nodes fail to sense the surrounding environment. As a result, some wrong data can be generated by these sensor nodes and the data can significantly decrease the accuracy of data fusion.

To solve the problems discussed previously, we introduce the reputation system into WSNs. The concept of reputation originated from sociology can be used to overcome the shortcomings of cryptography-based information fusion systems. Several reputation systems have been proposed in the literature [4,13]. In this paper, we will mainly pay our attention on the beta reputation system [13]. In beta reputation systems, reputation R_i,j_ is computed by sensor node N_i_ using beta density function of sensor node N_j_'s previous actions. For example, sensor node N_i_ counts the number of good and bad actions of N_j_ as r_i,j_ and s_i,j_. Then, N_i_ records the reputation R_i,j_ about node N_j_ as R_i,j_=Beta(*p*|r_i,j_ + 1,s_i,j_ +1) and the trust ${\text{T}}_{\text{i},\text{j}}=\text{E}\left({\text{R}}_{\text{i},\text{j}}\right)=\frac{{\text{r}}_{\text{i},\text{j}}+1}{{\text{r}}_{\text{i},\text{j}}+{\text{s}}_{\text{i},\text{j}}+2}$, where Beta represents the Beta distribution which can be expressed by the gamma function Γ as: Beat $\left(p|{\text{r}}_{\text{i},\text{j}}+1,{\text{s}}_{\text{i},\text{j}}+1\right)=\frac{\Gamma \left({\text{r}}_{\text{i},\text{j}}+{\text{s}}_{\text{i},\text{j}}+2\right)}{\Gamma \left({\text{r}}_{\text{i},\text{j}}+1\right)\Gamma \left({\text{s}}_{\text{i},\text{j}}+1\right)}p^{{\text{r}}_{\text{i},\text{j}}}{\left(1-p\right)}^{{\text{s}}_{\text{i},\text{j}}}$, where 0≤*p*≤1, r_i,j_, s_i,j_≥0.

In a cluster, every node records the reputation and trust values of other nodes and updates them periodically. In this way, the cluster head that executes the information fusion programs can have all the nodes' reputation clearly. In fact, the reputation is a small number ranging from 0 to 1. However, for convenience sake, we will transform the reputation of a sensor node into an integer from 0 to 10. Each normal sensor node needs to maintain a reputation table in which the reputation values of the neighbors are stored.

### 2.3. Secure Data Fusion in Wireless Sensor Networks

To our knowledge, secure data fusion in wireless sensor networks have not been widely researched, though there are several protocols recently proposed in the literature. Blind Information Fusion Framework (BIFF) is proposed in [14]. In BIFF, the data are transformed from normal space to the anonymous space and can't be deduced once they are fused. Another secure distributed data fusion system based on a consensus averaging method is proposed in [15]. Random Offset Method in [15] is composed of two steps, *i.e.*, obfuscate the fusion data by a noise process and recovery the obfuscating data by exploring high-frequency elimination property of consensus filter at fusion stage. A related problem is secure data aggregation in wireless sensor networks and it has been widely researched in [16,17].

## 3. Assumptions and Threat Model

In this section, we will introduce some assumptions and threat model. To defend against the threats, a simple mechanism is also presented at the end.

### 3.1. Assumptions

**Figure 1.**
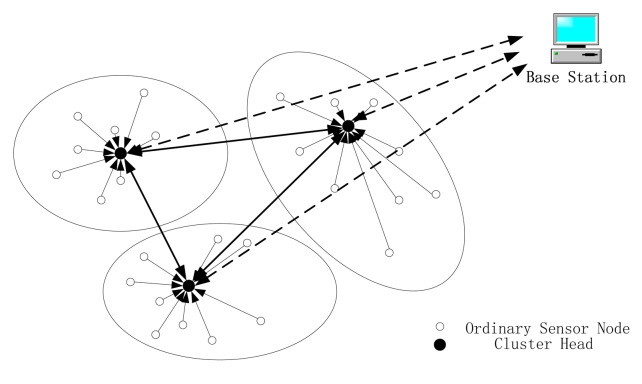
Topology of WSNs.

(a) We consider a WSN that composed of a large number of cheap sensor nodes. Because of the poor quality, the sensor nodes are highly likely to be compromised by the enemies. As a result, the enemies can get the secret keys through a physical way and cryptography alone can't guarantee the safety of the WSNs sufficiently.
(b) We assume that each sensor node can be selected as a cluster head and is capable of fusing data. The members of a cluster are stable unless some sensor nodes run out of energy. In this paper a randomized rotation of the high-energy cluster heads' positions among the sensors is designed to avoid draining the battery of any sensor in the network.
(c) We organize the WSN in a multi-tier hierarchical way, as shown in Figure 1. The sensor nodes are divided into several clusters and in each cluster they are controlled by a cluster head. Every sensor node can communicate with the members that in the same cluster and the cluster head. The cluster heads of the whole WSN comprise the second layer and they can communicate with each other. Custer heads can transfer information to the base station through a multi-hop manner. Besides, we assume that the base station, which is the bridge between the network and users, is secure all the time, *i.e.*, the base station can't be compromised. This is reasonable, because the quality of base station is much better than the sensor nodes.
(d) The same to [6], we assume that each sensor node has three keys, which are injected by the users: a master key, a cluster key and a pairwise key. The master key is shared by all the cluster heads, which facilitates the broadcast of the base station. The cluster key is unique for each cluster, which facilitates the communication from the cluster heads to the sensor nodes and group communication within a cluster. Node to node communication uses the pairwise keys. In this paper, we focus on describing the high level mechanism for protecting networks and therefore we do not consider the key distribution.
(e) In the initial stage, we assume that all of the sensor nodes are credible and as a result, the randomly selected cluster head is also credible. This is reasonable, because after being deployed, the sensor nodes begin to build a network at once and an attacker compromise the sensor nodes in such a short time is highly impossible.

### 3.2. Threat Model

(a) Traditionally, a compromised sensor node can attack the network in several ways, such as jamming, message dropping, information falsifying and so on. Besides the traditional attacks, in this paper, we also pay our attention on the following two attacks, which can significantly mislead data fusion results.
(b) Falsifying the local values: A compromised ordinary sensor node can falsify its own readings and upload the falsified data to mislead the data fusion results. We consider two manners of falsifying the data. Case (i): If there is only one compromised sensor node, the sensor node can falsify the data in a random way, *i.e.*, randomly generate the uploading data surrounding its own sensor reading with a distribution and variance. For example, when the sensor reading is 25 °C, the sensor can randomly generate the data with a Gaussian distribution with average 25 and variance 10 which can significantly decrease the stability of the data; Case (ii): If there are several compromised ordinary sensors in a cluster, they tend to cooperate with each other to maximize the misleading effect on data fusion result. For example, the compromised sensors can make the normal data larger or smaller together rather than some of them do. In this way, data fusion results can be affected significantly.
(c) Falsifying the fusion results: In a cluster, if the cluster head is compromised, the cluster head can attack the network by falsifying a data fusion result and upload the falsified result to the base station. It is obvious that falsifying the fusion result is a more serious attack compared with falsifying the local value. It is a big challenge to guard against this attack. However, we can address this attack by employing DCHM.

**Figure 2.**
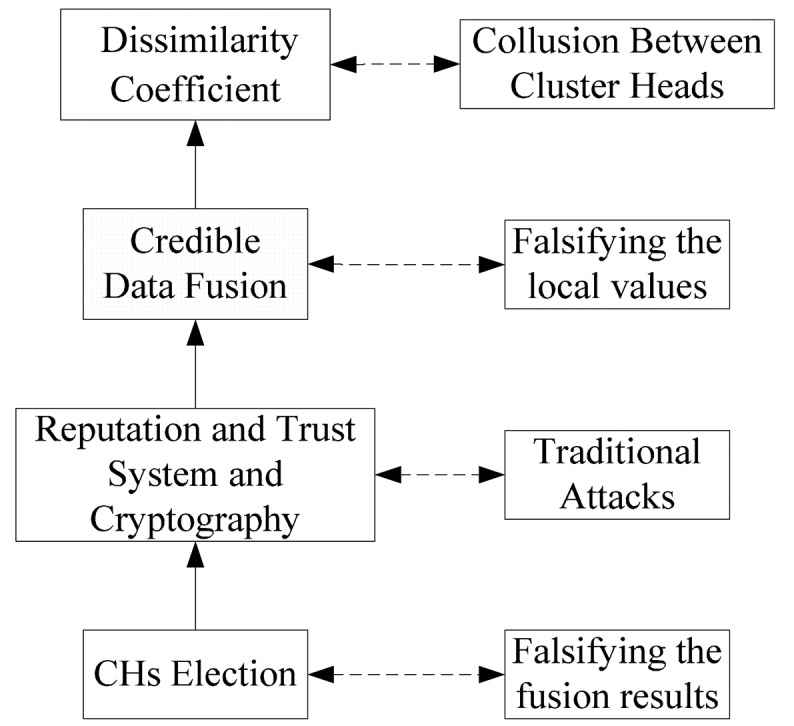
A brief mechanism to defend against threats.

Figure 2 gives a brief mechanism to defend against these threats effectively. In this mechanism, several techniques are employed and each technique is capable of defending a specific aspect of threat model. Intuitively, incorporating them together properly is a good solution. In fact, DCHM is a more complete version of the brief mechanism and we will introduce DCHM in the next section in detail.

## 4. Double Cluster Heads Model for Data Fusion

### 4.1. Overview of the Framework

**Figure 3.**
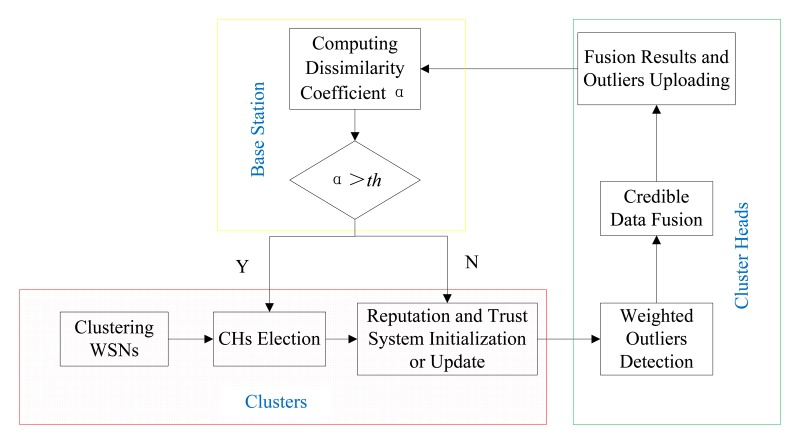
Overview of the Framework.

As shown in Figure 3, the whole framework of DCHM is composed of three modules, *i.e.*, Cluster Module, Cluster Heads Module and Based Station Module. Cluster Module includes three sub-modules, *i.e.*, clustering WSNs, Cluster Heads Election, Reputation and Trust System Construction or Update. Cluster Heads Election also includes three sub-modules, *i.e.*, Weighted Outliers Detection, Credible Data Fusion, Fusion Results and Outliers Uploading. The function of base station part is judging the security of the cluster heads based on the uploading information. There is a feedback from base station part to cluster part and the feedback makes contributions to improve the security of the cluster heads and accelerating the convergence of the reputation and trust systems.

The DCHM is divided into three modules, because each module is performed in a particular position of the network and act as a particular role. In Clusters Module, each sensor node in a cluster needs to participate in the processes of clustering, cluster heads election and constructing and updating the reputation and trust system. This module is the basement of whole framework and the information collected by the sensor nodes in a cluster is transmitted to the cluster heads. Cluster Heads Module is a bridge between Clusters Module and Base Station Module. Cluster Heads Module is performed by the two cluster heads and has two responsibilities, *i.e.*, outlier detection and credible data fusion. Cluster heads can guarantee the credibility of the sensor nodes in a cluster and its credibility needs to be checked by the Base Station Module. Based on the fusion results and the lists of outliers uploaded by the cluster heads, the base station establishes feedback to the Clusters Module which can help the clusters to update the reputation and trust systems. Note that, it is assumed that the base station is credible all the time. The three modules are integrated together to protect the network and can't be separated from each other. We introduce each module of the framework in the following.

### 4.2. The Cluster Module

#### 4.2.1. Clustering and Cluster Heads (CHs) Election

In the initial phase, we divide the sensor nodes into several clusters by an arbitrary clustering algorithm, such as LEACH-C [11]. After clustering, two random sensor nodes in a cluster are selected as the cluster heads and, as discussed previously, the cluster heads are assumed to be trusted. Then, each sensor node in a cluster begins to monitor the surrounding environment and record the monitoring data. Meanwhile, each sensor needs to monitor the immediate neighbors' behaviors and maintain a trust table in which the reputation of the neighbors is recorded in detail.

**Figure 4.**
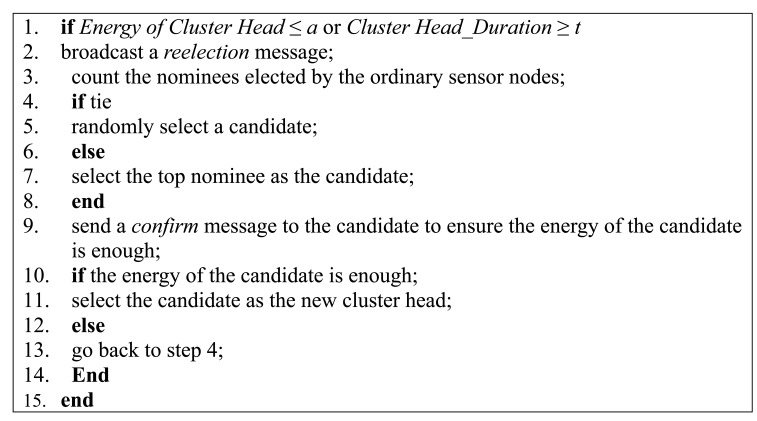
Procedure performed by cluster head.

**Figure 5.**
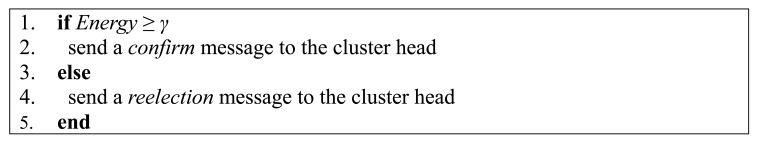
Procedure performed by the candidate.

**Figure 6.**
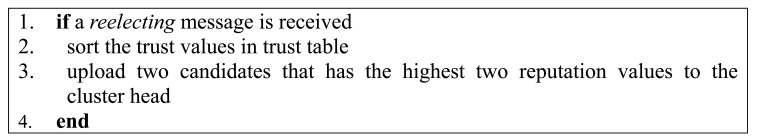
Procedure performed by ordinary sensor nodes.

A cluster head needs to be reelected under two circumstances: first, the residual energy of the cluster head falls below a threshold, *a*, which is preset by the users; second, the cluster head have served for a period of time which is longer than another preset threshold, *t*. In the simulation part, *a* is set to 0.001 J and *t* is set to 100 s. In the process of cluster head election, cluster head, the candidate and ordinary sensor nodes have different actions and a high level description of these actions are shown in Figures 4, 5 and 6, respectively.

The cluster head first broadcasts a reelection message in a cluster. Then, all the sensor nodes in the cluster vote for a new cluster and two candidates are uploaded by each sensor node. The current cluster head tallies the votes and decides the winner based on majority rule. After a sensor node being selected as a candidate, a *confirm* message is sent to the sensor node to ensure that the energy of the candidate is enough. However, if the energy of the candidate is not enough, the current cluster head need to select a new candidate until a candidate with enough energy is selected and this candidate is the new cluster head.

If a sensor node is selected as a candidate, it needs to response *confirm* message based on its energy. If its energy is enough, a *confirm* message is sent to the cluster head; otherwise, a *reelection* message to the cluster head. Note that, a redundant cluster head, called vice cluster head, is on standby in case of emergency. Vice cluster head performs like normal sensor nodes when the cluster head works normally. However, when a cluster head breaks down suddenly, the vice cluster head needs to replace the cluster head to fuse data. Another problem is how to ensure the condition of vice cluster head. To solve this problem, the vice cluster head needs to send a *normal condition* message to the cluster head periodically. If the energy of vice cluster head falls below a threshold *γ*, which is preset by the users, a *reelection vice cluster head* message is uploaded to the cluster head. Specially, we set *γ* to be 0.001 J, which is one-tenth of the initial energy. The procedure of reelecting a vice cluster head is the same with a cluster head. On the other hand, if the vice cluster head breaks down suddenly and the *normal condition* message cannot be uploaded periodically, the cluster head needs to start up the procedure of reelecting a vice cluster head.

After receiving the reelection message, the ordinary sensor nodes vote for a candidate. Each sensor node needs to sort the trust values in its trust table and vote for two neighbors that have the highest two trust values. The *voting* message needs to be encrypted with the cluster key.

#### 4.2.2. Reputation and Trust System Initialization or Update

The initialization of the reputation and trust system is the same to traditional ones [4] and we focus on updating our system. Based on the relation of *α* and th which will be discussed in Section 4.4, the system updates in two ways.

If the original cluster heads are credible, an outlier list is sent to the cluster and each sensor node needs to compare the list with its own trust table and the outliers' trust value *Tru* decreases by a parameter *β* which will be discussed in Section 4.4 in detail.

If the original cluster heads are incredible, only the cluster heads' trust value is set to 0.

### 4.3. The Cluster Module

#### 4.3.1. Outlier Detection Based on Weighted DBSCAN Algorithm

DBSCAN algorithm [18] is one of the famous density-based clustering algorithms in data mining and it can be used to detect outliers. However, we need to revise DBSCAN slightly to apply it to weighted data outliers detection. First, some revised definitions are briefly introduced and the detailed presentation can be found in [18].

**Definition 1**: (*Rad* -Neighbor of a point) The *Rad*-Neighbor of a point *q*, denoted by *Nei_Rad_(q)*, is defined by *Nei_Rad_(q)* = {*p*∈*S* | *dist(p,q)* ≤ *Rad*},where *S* is the set of all the data points.

In reputation and trust-based sensor networks, each data object has a weight, *i.e.*, the trust. We revise the definition of *directly density-reachable* as follows.

**Definition 2**: (directly density-reachable) A point *p* is directly density-reachable is from a point *q* with regard to *Rad* and *MinPts* if:

(1) *p* ∈*Nei_Rad_(q)* and
(2) *Wei(Nei_Rad_(q))* ≥ *MinPts*,

where *Wei(Nei_Rad_(q))* is the total weight of all the neighbors of data point *q*.

**Definition 3**: (*density-reachable*) A point *p* is *density-reachable* from a point *q* with regard to *Rad* and *MinPts* if there is a chain of points *p_1_*,…,*p_n_, p_1_* = *q, p_n_* = *p* such that *p_i_*_+_*_1_* is directly density-reachable from *p_i_*.

**Definition 4**: (*density-connected*) A point *p* is *density-connected* to a point *q* with regard to *Rad* and *MinPts* if there is a point *o* such that both *p* and *q* are *density-reachable* from *o* with regard to *Rad* and *MinPts*.

**Definition 5**: (cluster) Let *S* be a set of monitoring data *X*. A cluster *C* with regard to *Rad* and *MinPts* is a non-empty subset of *S* satisfying the following conditions:

(1) ∀*p,q*: if *p*∈*C* and *q* is density-reachable from *p* with regard to *Rad and MinPts*, then *q*∈*C*.
(2) ∀*p,q*∈*C*: *p* is density connected to *q* with regard to *Rad* and *MinPts*.

**Definition 6**: (credible data) There may be several clusters in the set of data and each cluster contains some data points with different reputations. The data in the cluster that has the highest total weight are credible data.

In this paper, Euclidean distance is adopted to measure the similarity between two data points when using DBSCAN algorithm to detect the outliers. A problem of Euclidean distance is that some feathers with large amplitude often cover the effect of other feathers with small amplitude. Therefore, a preprocessing is used to normalize the original *n* data points in data set *X*:

(1) $$x_{i}^{j}=\frac{x_{i}^{j}-x_{\text{min}}^{j}}{x_{\text{max}}^{j}-x_{\text{min}}^{j}}$$

where $x_{i}^{j}$ is the *j* th feather of data point *x_i_*, $x_{\text{min}}^{j}=\text{min}\left(x_{1}^{j},x_{2}^{j},\ldots ,x_{n}^{j}\right)$, $x_{\text{max}}^{j}=\text{max}\left(x_{1}^{j},x_{2}^{j},\ldots ,x_{n}^{j}\right)$. The distance between data points *x_i_* and *x_j_* is defined by:

(2) $$\text{dist}\left(x_{i},x_{j}\right)=\text{sqrt}(\sum {\left(x_{i}^{d}-x_{j}^{d}\right)}^{2})$$

where *d* is the dimension of the data points. After detecting the outliers, the normalized data points need to be restored by:

(3) $$x_{i}^{j}=x_{i}^{j}*\left(x_{\text{max}}^{j}-x_{\text{min}}^{j}\right)+x_{\text{min}}^{j}$$

In most cases, there is only one cluster in which the data are credible and other data are outliers. However, if the amount of compromised sensor node is very large, there may be several clusters and we can choose the credible data by definition 6. Based on the definitions presented previously, the DBSCAN algorithm can be directly used to detect the outliers in weighted data set. A challenge is to choose proper parameters *Rad* and *MinPts*. Fortunately, similar with the conclusion in [18], we find that *MinPts* can be set to 3 and we can always get a good result. As a result, *Rad* is determined by *MinPts* of the method in [18] and in simulation part, *Rad* is set to 0.2.

Haven detected the outliers, the cluster heads need to use a list, called *L*, to record all these outliers and upload the list to the base station. Meanwhile, the data fusion result, called *R*, is also uploaded and data fusion is presented in following.

#### 4.3.2. Credible Data Fusion

After getting the credible data, we need to fuse them by a proper data fusion method. In this paper we fuse them by Bayesian data fusion. It is reasonable that the fusion results of the credible data are more accurate than the original data, since the outliers have been deleted.

Bayesian data fusion enables fusion of pieces of data and lies at the core of probability based data fusion algorithms. We assume a state-space representation *X* which is the range of the fusion results, the Bayesian data fusion algorithm provides a method for computing the posterior conditional probability distribution/density of the hypothetical state x_k_ given the set of measurements Z = {z_1_, z_2_,…,z_n_}, where n is the number of sensor nodes whose readings need to be fused and the prior distributions, as following:

(4) $$\text{p}\left({\text{x}}_{\text{k}}|\text{Z}\right)=\frac{\text{p}\left({\text{x}}_{\text{k}}\right)*\text{p}\left(\text{Z}|{\text{x}}_{\text{k}}\right)}{\text{p}(\text{Z})}$$

where p(Z∣x_k_) is called the likelihood function and based on the given sensor measurement model, p(Z) is a constant and it is merely a normalization term to ensure that the probability density function is integrate to one. We maximize p(x_k_|Z) to get x_k_ which is the data fusion result. Specially, in our approach, Z is the set of the temperatures need to be fused and p(Z|x_k_) is decided by the property of the sensor nodes. In addition, we set p(x_k_) to a uniform distribution in the range of possible temperatures.

Note that, both data fusion result and outliers list are needed to upload to the base station and they are used to compute the dissimilarity coefficient.

### 4.4. The Base Station Module

The main function of Base Station Module is computing Dissimilarity Coefficient, denoted by *a*, and comparing *a* with a threshold *th*, which is preset by users. We define *a* based on both two data fusion results *R_1_, R_2_* and the outlier lists *L_1_, L_2_* as follows:

(5) $$\alpha =\varepsilon_{1}*\frac{2*\left|R_{1}-R_{2}\right|}{\left|R_{1}+R_{2}\right|}+\varepsilon_{2}*(1-\frac{\left|L_{1}\cap L_{2}\right|}{\left|L_{1}\cup L_{2}\right|})$$

where *ε_1_* and *ε_2_* are the weights preset by the users and the sum of them equals to 1. *|L_1_*∩*L_2_*| is the number of the common outliers in *L_1_* and *L_2_*. *|L_1_*∪*L_2_*| is the number of all the outliers in *L_1_* and *L_2_*. The threshold *th* is another parameter preset by the users. In simulation part, *ε_1_* and *ε_2_* are both set to be 0.5 and *th* is set to be 0.2. It is obvious that, if the two cluster heads are both credible, *α* would be smaller than a constant value *th* and vice verse. After checking whether the cluster heads are credible, a feedback loop is designed to reelect the cluster heads or to decrease the trust values of the outliers.

**Figure 7.**
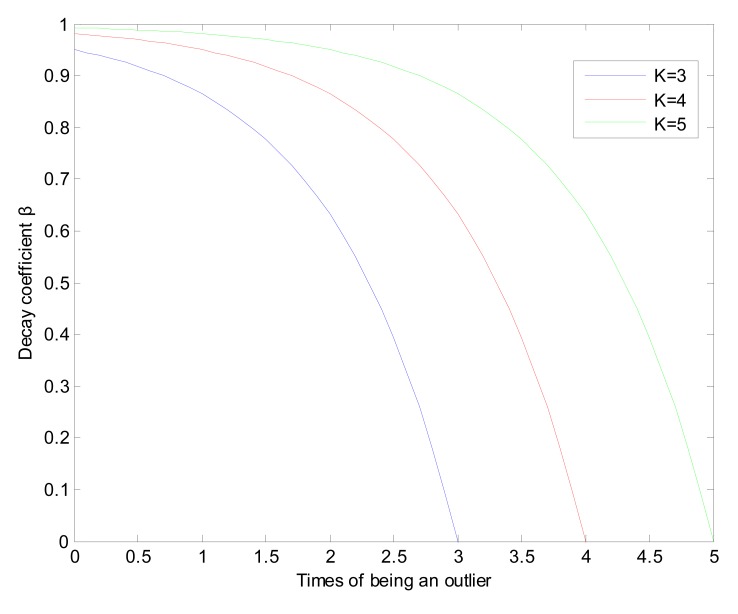
Decay function *β*.

If *α* ≤ *th*, which means that both the cluster heads are credible, only the reputation and trust system should be updated. The base station sends the list of *|L_1_*∩*L_2_*| to the cluster and each of the sensor nodes in the cluster needs to check whether its trust table contains the outliers or not. If a outlier is contained in the trust table, the trust of the outlier decreases to Tru*β, where 0 ≤ *β* < 1. In this paper, *β* is defined as follows:

(6) $$\beta =\{\begin{array}{c} 1-e^{T-K},T\leq K\\ 0,T>K \end{array}$$

where *K* is a parameter which can control the rate of change of *β* and *T* are the times that a sensor node's monitoring data is detected as an outlier. In the simulation part, *K* is set to be 5. As shown in Figure 7, with the increasing of *K*, the change rate of *β* decrease. Another property is that when *T* = *K, β* decreases to 0.

If *a* > *th*, two temporary cluster heads in the cluster need to be reassigned by the base station and then two new cluster heads need to be reelected as discussed in Section 4.2. In the process of reputation and trust system updating, the original cluster heads' trust values are set to 0.

**Figure 8.**
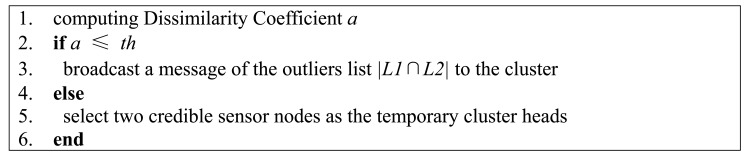
The procedure performed by the base station.

When a sensor node's trust value is 0, there will be a black hole phenomenon, *i.e.*, all the neighbors will not cooperate with the sensor node. As a result, the sensor node with trust value 0 can do nothing to attack the network. A pseudo-code of the feedback loop is shown in Figure 8.

## 5. Performance Evaluations

### 5.1. Simulation Setup

We simulate our model based on the ns-3 (version ns-3.21) simulator [19]. In order to study the performance of DCHM, we consider a cluster based WSN to monitor the fire in a terrain. To recognize the fire, we need to record the temperature and humidity simultaneously. We assume that each sensor node is capable of detecting both the temperature and humidity. For our basic simulation network topology, 150 sensor nodes are scattered in a 400 m × 400 m square area randomly and they are divided into three clusters by LEACH-C [11]. The base station is located at the center of the square area and it is connected with the external network. Each node has a radio range of 50 m.

In our simulation, the ns-3 simulator implements a 1 Mb/s 802.11 MAC layer. The length of message without using DCHM is always 24 bytes. However, in the networks using DCHM, each data message transmitted from an ordinary sensor node to the cluster heads is 27 bytes long. The length of the messages transmitted from cluster heads to the base station and transmitted from the base station to the ordinary sensor nodes depends on the number of outliers. There are three modes for a sensor node, *i.e.*, sending message, receiving message and sleeping mode. As in [11,20], the energy consumption for sending a message is given by *l* × *E_elec_* + *l* × *ε* × *d^2^*, and for receiving a message, the energy consumption is *l* × *E_elec_*, where *l* is the length of a message, *d* is the distance of message transmission, *E_elec_* is set at 50 nJ/bit. As in [20], in our simulations, each sensor node begins with only 0.01 J of energy. In our simulation, the data packets are generated every five seconds and the whole framework is updated meanwhile.

In our simulation, the time that a compromised sensor node *csn_i_* acts as a cluster head is denoted by *t_i_* and the probability of selecting compromised sensor nodes as cluster heads is defined as follows:

(7) $$\text{P}=\frac{\sum_{i=1}^{nc}t_{i}}{2*\text{Life}}$$

where *nc* is the number of compromised sensor nodes, *Life* is the lifetime of the whole network. Note that, the whole network is out of use when at least one sensor node in a cluster does not work. For example, the lifetime of a network is 10,000 s and in cluster 1, there is no cluster head acted by a compromised sensor node, in cluster 2, a compromised sensor node acts as a cluster head for 100 s, and in cluster 3, one cluster head is acted by a compromised senor node for 500 s and the other cluster head is acted by a compromised senor node for 400 s, then P equals to $\frac{100+500+400}{2*10000}=5\%$.

As discussed previously, the compromised sensor nodes can attack the network in several traditional ways, such as jamming, message dropping, information falsifying and so on. However, for convenience, only message dropping is considered in this paper and message drop ratiois 50% for a compromised sensor node and 10% for a credible sensor node. Besides, we pay our attention on another two threats, *i.e.*, falsifying the local values and falsifying the fusion results. As shown in Table 1, the sensor node measurement model is set to be a Gaussian distribution with mean value *Mean* and standard deviation *Std*. For a credible sensor node, *Mean* is set to the real environment value and *Std* is set to 2. For a compromised sensor node, to mislead data fusion results, *Mean* equals to 1.1 times of the real monitoring value and *Std* is set to 4.

Our reputation and trust system is compared with the system in [4] especially in the aspect of convergence time. We randomly choose a credible sensor node and monitor its trust table and present the changes of trust values.

At last, we simulate the accuracy of data fusion based on our model. The temperature in the terror is set to be 25 °C and the relative humidity is 25%. We mainly compared our result directly with data fusion result without any protection mechanism.

Note that, every simulation discussed previously is done for 100 times and lasts for 1000 s, *i.e.*, 200 packets are generated for each sensor node. We present the average results of the experiments in the next section and a detailed discussion is also described.

### 5.2. The Security of Data Fusion Results

In this section, we demonstrate the security of data fusion results from two aspects, *i.e.*, the probability of selecting compromised sensor nodes as cluster heads and the evolution of trust values for credible sensor nodes and compromised sensor nodes, respectively.

**Figure 9.**
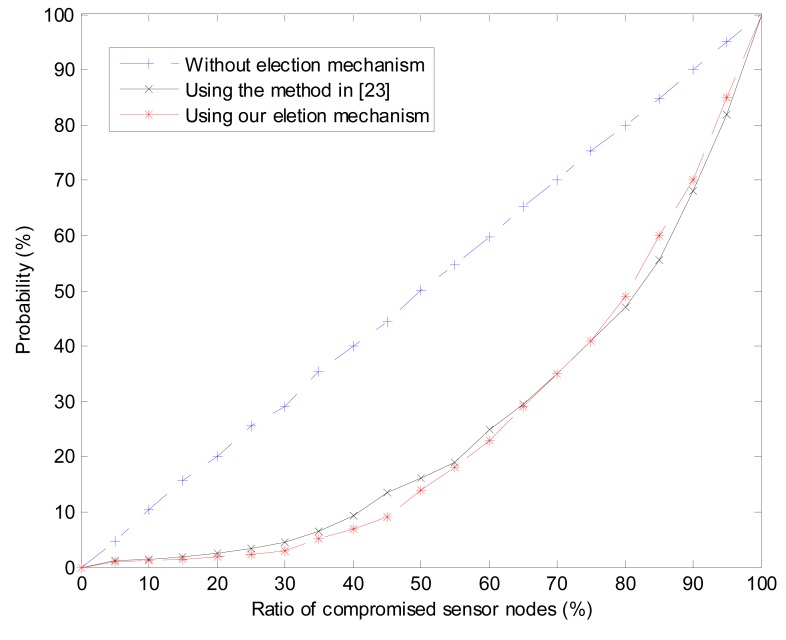
Probability of selecting compromised sensor nodes as cluster heads.

When simulating the probability of selecting a compromised sensor node as a cluster head, the ratioof compromised sensor nodes, denoted by radio, ranges from 0% to 100% with intervals of 5%. We compared our cluster heads election mechanism with trust-based election mechanism (TEM) in [6]. As shown in Figure 9, if the cluster heads are randomly selected without any mechanism, the probability of choosing compromised sensor nodes linearly increase with the increasing of ratio. Both the TEM and our mechanism perform very well especially when radio ⩽ 15%. When 15% ⩽ radio ⩽ 65%, our cluster heads election mechanism performs slightly better than TEM and when radio ≥ 75%, TEM performs slightly better. This phenomenon can be explained that if compromised sensor nodes are much more than the credible sensor nodes, the feedback loop in our model is counterproductive. The performance of our mechanism is acceptable because, in most cases, it is impossible that the compromised sensor nodes are much more than the credible ones.

**Figure 10.**
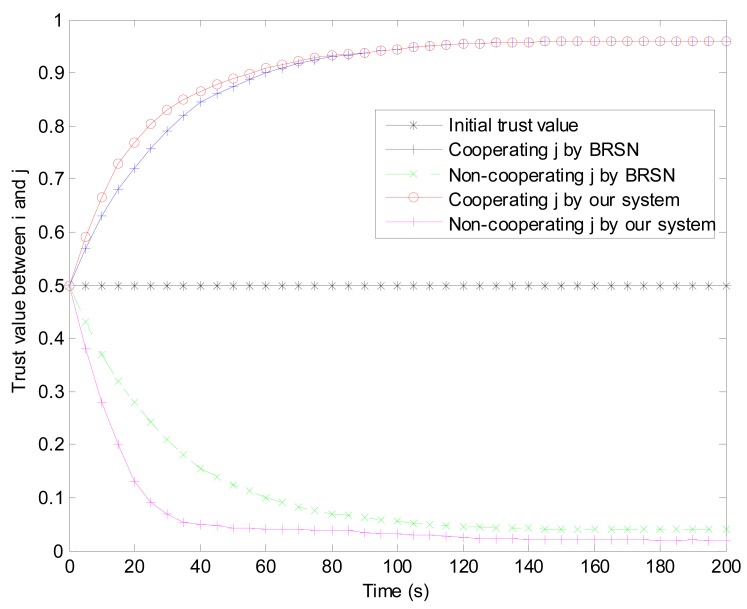
Evolution of j's trust values in sensor node i.

As shown in Figure 10, the trust value for a credible sensor node increases monotonously to an upper limit. On the contrary, a compromised sensor node's trust value decreases monotonously to a lower limit. We also compare the performance of our reputation and trust system against BRSN in the aspect of convergence time. In initial stage, the trust value of j in i is set to be 0.5 because sensor node i can't detect whether j is credible or not. The trust value updates and becomes stable as time goes on. If j is credible and cooperates with i, the trust value becomes stable in about 100 s and our system's convergence time is shorter. If j is a compromised sensor nodes, its trust value becomes stable in about 100 s by BRSN. However, in our reputation and trust system, the convergence time is about 35 s which is much less than 100 s. Simulation illustrates that our reputation and trust system performs much better than BRSN in convergence time especially for a compromised sensor node.

### 5.3. The Accuracy of Data Fusion Results

**Figure 11.**
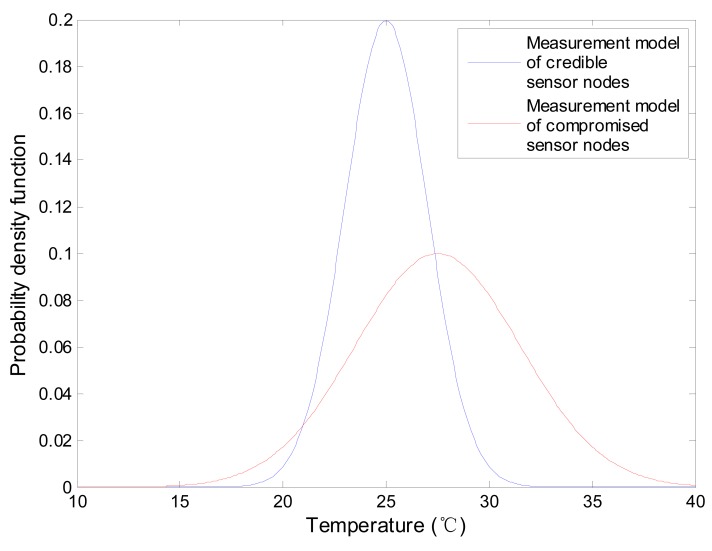
Measurement models of sensor nodes.

This section investigates the accuracy of DCHM-based data fusion algorithm. The comparisons between data fusion algorithm without DCHM and using DCHM are presented. As discussed previously, the measurement models of sensor nodes are Gaussian distributions and they are shown in Figure 11. The mean values of the two measurement models are 25 and 27.5, respectively. Obviously, the compromised sensor nodes can significantly mislead the data fusion results.

**Figure 12.**
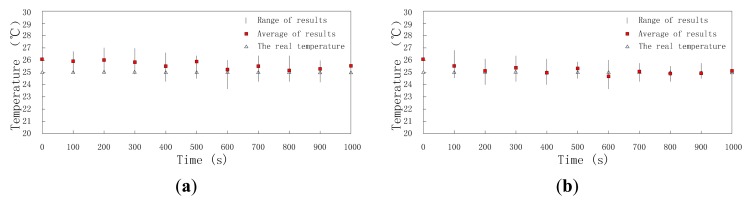
Data fusion results of temperature *versus* time. (**a**) Without DCHM; (**b**) Using DCHM.

**Figure 13.**
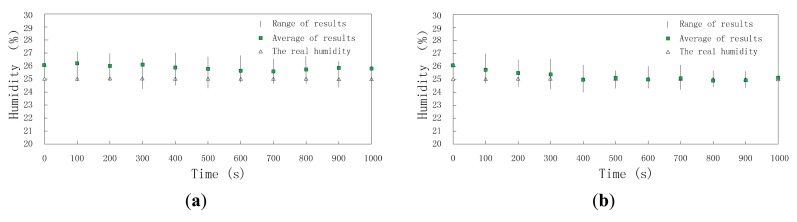
Data fusion results of humidity *versus* time; (**a**) without DCHM and (**b**) using DCHM.

Data fusion results are affected by time and the ratio of compromised sensor nodes. On one hand, when ratio is a constant value, data fusion results change with time, because the reputation and trust system updates as time goes on; on the other hand, at a constant time, data fusion results are different because of different ratios and the higher the ratio is, the worse the data fusion result is. First, we set ratioto 30% and research the change of data fusion results with time. Though the real temperature and humidity are assumed to be stable all the time, the data fusion results change as time goes on as shown in Figures 12 and 13. When the clusters don't employ DCHM as shown in Figures 12a and 13a, the data fusion results are always higher than the real value. Without DCHM, the errors of data fusion results, *i.e.*, the distances between the squares and triangles are random. This phenomenon illustrates that the data fusion results are misled by the compromised sensor nodes and as a result, the users of WSNs are also misled. On the contrary, when the clusters employ DCHM, the data fusion results are much better as shown in Figures 12b and 13b. In the initial stage, data fusion results are higher than the real value, because the misleading effects of compromised sensor nodes. However, as time goes on, data fusion results becomes close to the real value and about 400 s later, data fusion results are stable and the error is very little.

**Figure 14.**
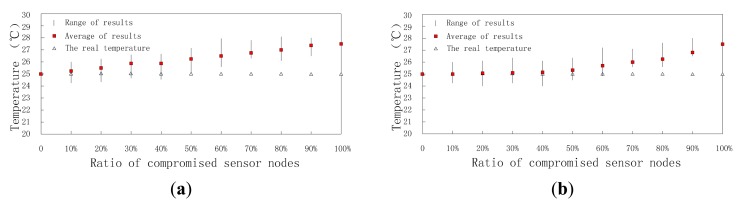
Data fusion results of temperature *versus* ratio; (**a**) without DCHM and (**b**) using DCHM.

**Figure 15.**
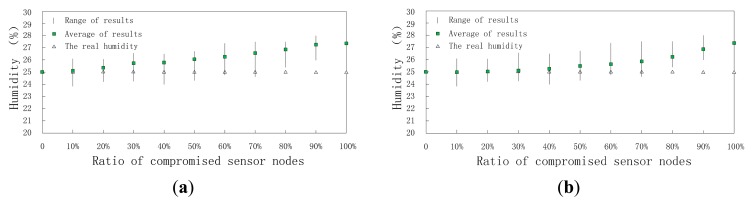
Data fusion results of humidity *versus* ratio; (**a**) without DCHM and (**b**) using DCHM.

We also research how the rations of compromised sensor nodes affect the data fusion error. In this experiment, we set time to the 300-th second and ratio ranges from 0% to 100% with intervals of 10%. The results are shown in Figures 14 and 15.

As shown in Figures 14a and 15a, without DCHM, data fusion results go away from the real value with a linear speed. That is to say the data fusion errors are affected by the compromised sensor nodes directly. However, when using DCHM, the errors increase in a mild way. The accuracy of data fusion results improve significantly when 20% ≤ radio ≤ 70%. When radio ≤ 20%, both of them perform well and when radio ≥ 80% data fusion results are meaningless to the users.

### 5.4. Average Energy Consumption and Network Lifetime

The average energy consumption measures the ratioof total dissipated energy of the whole network in a second to the number of all the sensor nodes. As declared in Section 5.1, DCHM updates once every five seconds. We first give the simulation result of the average energy consumption over the radio range of the sensor nodes and then present the lifetime of the networks with and without DCHM, respectively.

**Figure 16.**
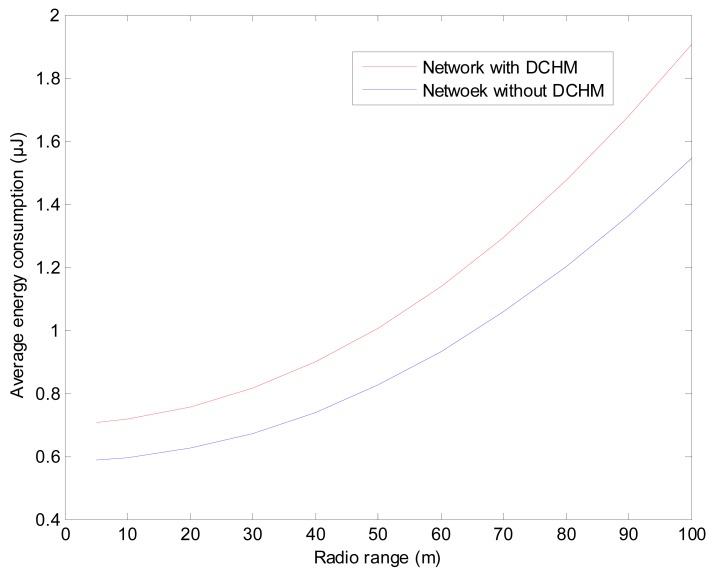
Average energy consumptions *versus* radio range of a sensor node.

In order to make the sensor nodes can communication with its neighbors with a constant radio range, we need to scale the size of the network and ensure that most of the sensor nodes can communicate with three to seven neighbors. Figure 16 presents the simulation result and we can find that the average energy consumption of the network with DCHM is always larger than that of the network without DCHM. This is reasonable, because in the network with DCHM, some extra data is computed, stored, transmitted and received. In conclusion, the average energy consumption in networks with DCHM is about 1.2 to 1.3 times that in networks without DCHM. As a result, the lifetimes of the networks are also very different.

**Figure 17.**
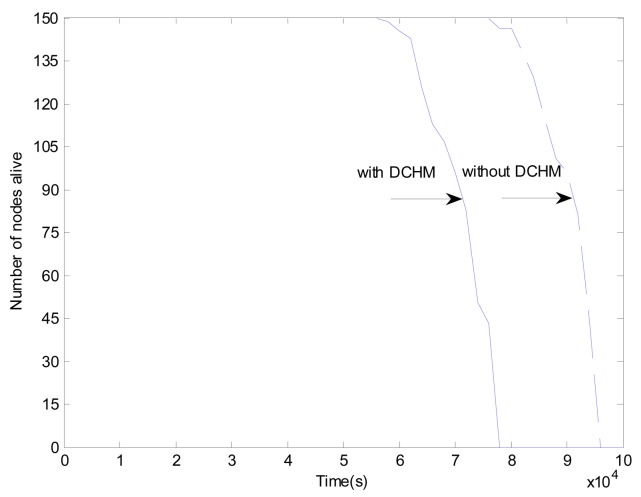
Number of nodes alive over time.

Figure 17 shows the number of nodes alive over time in the networks with and without DCHM, respectively, when the radio range is 50 m. We can find that in the first 5.5 × 10^4^ s, most of the nodes are alive in both networks. However, the number of the sensor nodes alive in the network with DCHM begins to decrease significantly after 5.5 × 10^4^ s and that in the network without DCHM begins to decrease significantly after 7.5 × 10^4^ s. The nodes in the network without DCHM remain alive for a longer time, because a smaller amount of data has been processed in the network without DCHM. In conclusion, the DCHM is a tradeoff between security and energy efficiency of the networks.

## 6. Conclusions

In this paper, we discussed the problem of secure and accurate data fusion in WSNs. We attempt to solve the problem by integrating clustering techniques, reputation and trust systems, and data fusion algorithms with each other. To our knowledge, it is the first time to solve the problem by this way. We discussed how to avoid selecting the compromised sensor nodes as cluster heads, detect the outliers and update the reputation and trust systems. These sub-modules comprise the whole DCHM which can defend against the attacks presented in Section 3. Besides the traditional attacks, two new attacks, which are designed specially to mislead the data fusion results, are also considered. Meanwhile, the accuracy of data fusion results is also improved because the compromised sensor nodes are detected and eliminated in the networks. Simulation shows that our model performs well in improving the security and accuracy of data fusion. Besides, the convergence time of the reputation and trust system becomes much shorter. For future work, we plan to design a more intelligent mechanism to decide the parameters which is some difficult for the users to preset. This mechanism would guarantee the versatility of our model in any cases.

## Figures and Tables

**Table 1.** Comparisons between credible sensor nodes and compromised sensor nodes.

**Types of Sensor Nodes**	**Message Dropping Ratio**	**Measurement Model**	***Mean***	***Std***
Credible sensor nodes	5%	Gaussian distribution	The real environment value	2
Compromised sensor nodes	50%	Gaussian distribution	1.1 times of the real environment value	4
